# Diffusion-Weighted Imaging with Two Different *b*-Values in Detection of Solid Focal Liver Lesions

**DOI:** 10.1155/2016/8128207

**Published:** 2016-02-25

**Authors:** Da-wei Yang, Ke-yang Wang, Xun Yao, Hui-yi Ye, Tao Jiang, Yuan Liu, Jia-yin Gao, Min Chen, Cheng Zhou, Zheng-han Yang

**Affiliations:** ^1^Department of Radiology, Beijing Friendship Hospital, Capital Medical University, Beijing 100050, China; ^2^National Clinical Research Center for Digestive Diseases, Beijing 100050, China; ^3^Department of Radiology, Peking University People's Hospital, Beijing 100044, China; ^4^Department of Radiology, PLA General Hospital, Beijing 100853, China; ^5^Department of Radiology, Beijing Chaoyang Hospital, Capital Medical University, Beijing 100020, China; ^6^Department of Ultrasound, Beijing Hospital, Beijing 100730, China; ^7^Department of Radiology, Beijing Hospital, Beijing 100730, China

## Abstract

One hundred and eighty-two consecutive patients with suspected liver disease were recruited to receive diffusion-weighted imaging (DWI) with two different *b*-values, in comparison with T2-weighted imaging (T2WI). The detection rate of three MR sequences in solid focal liver lesions (FLLs) and subgroup analyses were performed. Our prospective study found that DWI600 was equivalent to DWI100 and T2WI for the detection of solid FLLs overall but was significantly more accurate in the detection of malignant solid FLLs and lesions larger than 10 mm.

## 1. Introduction

Early detection and diagnosis of hepatic tumor are an important step in clinical work, which would allow effective surgical or mini-invasive therapy [1–6]. With the advances in magnetic resonance imaging (MR) technology, diffusion-weighted magnetic resonance imaging (DWI) is now widely used as a standard imaging sequence in clinical work and shows its potential benefit in evaluation of the focal hepatic tumor [7–11]. DWI with small *b*-values less than 100–150 sec/mm^2^ can suppress the intrahepatic vascular signal, creating the so-called black blood effect, which improves the detection of small focal liver lesions (FLLs) especially localized near small hepatic vessels. Meanwhile, DWI with low *b*-value has higher imaging quality compared with single shot fast spin-echo sequences [11–13], due to the fact that it is less affected by artifacts such as eddy currents or blurring.

A substantial number of studies [14–17] have compared low *b*-value DWI with T2-weighted imaging (T2WI) for image quality and detection of FLLs. These studies generally showed better performance of DWI with low *b*-value in terms of lesion detection and conspicuity compared with T2WI. However, most previous studies mainly focused on the detection of metastases or just included cysts and hemangiomas as benign lesions, lacking of common solid FLLs such as focal nodular hyperplasias (FNHs), or other less common solid FLLs such as inflammatory pseudotumors (IPTs). Furthermore, DWI with low *b*-value could not simultaneously improve the detection as well as characterization of lesion, which is usually performed with DWI with higher *b*-value (*b* > 500 sec/mm^2^) and/or other conventional sequences.

DWI with higher *b*-value mainly reflects diffusion information of water molecules motion within the lesions, which help to improve the characterization of solid FLLs [8]. Meanwhile, we found in practice that DWI with higher *b*-value also enables a better detection of lesions in liver or pancreas compared with T2WI or other conventional sequences. For example, solid FLLs such as FNHs and hepatocellular carcinomas (HCCs) sometime can be difficult to be detected on T2WI or even DWI with low *b*-value due to either iso- or slightly hypersignal intensity to liver parenchyma [18–20]; however, those lesions could be more conspicuous on DWI with higher *b*-value. Although some studies [16, 21–23] have investigated DWI with higher *b*-value in FLL detection, none of the studies has discussed the role of DWI with higher *b*-value in detection of solid FLLs covering the topic of common disease. Therefore, the purpose of this multicenter clinical study was to prospectively investigate the DWI with low *b*-values of 0, 100 sec/mm^2^(DWI100), DWI with higher *b*-value of 0, 600 sec/mm^2^ (DWI600) in detection of solid FLLs in a large number of patients with a wide spectrum of lesions, in comparison with T2WI.

## 2. Methods and Materials

### 2.1. Patients

This prospective, multicenter study was approved by Institutional Human Ethics Board and registered in http://www.chictr.org.cn/ numbering ChiCTR-DDT-11001587. Written informed consent was obtained from all patients. From June 2011 to December 2012, 182 patients were recruited in 6 hospitals (Beijing Friendship Hospital, Beijing Hospital, PLA General Hospital, Beijing Chaoyang Hospital, Nantong Third People's Hospital, and Ningbo Lihuili Hospital). The inclusion criteria were patients who have (1) focal liver lesions (FLLs) found on ultrasound or CT, (2) chronic liver disease or cirrhosis or viral hepatitis B/hepatitis C infection, (3) extrahepatic malignancy, and (4) no contraindication to MR contrast agents. 85 patients were excluded according to at least one of the following exclusion criteria: (1) undergoing antineoplastic treatment before MR scanning (*n* = 8), (2) no definite diagnosis (*n* = 6), (3) incomplete DW imaging (*n* = 3), (4) no lesion detected (*n* = 37), and (5) only cystic lesion detected (*n* = 31). Those without histologic diagnosis as well as typical MR findings finally were defined as patients with no definite diagnosis, who were then strongly suggested to take follow-up under the guidance of doctor considering other useful clinical tests. Patients with incomplete DW imaging were excluded from the study population; however, the lack of several DW images did not hamper making diagnosis.

### 2.2. MR Imaging

A 1.5 T MRI whole-body scanner (Signa Twin-speed HD, GE Healthcare, Milwaukee, WI) with an eight-element phased array coil was used for signal reception in all study sites. Gradient strengths were 23/40 mT/m. Gradient slew rate were 80/150 mT/m/ms. Diffusion-weighted MR imaging with low and higher *b*-value sequences and respiratory-triggered T2-weighted fast spin-echo imaging were performed as study MR sequences. The spectrally selective fat suppression technique was used at all three MR sequences. The detailed parameters for three MR sequences are shown in Table 1. We choose the *b*-value of 100 s/mm^2^ and 600 s/mm^2^ to represent the low and higher *b*-value, respectively, because both of them were used routinely in our clinical work and proven to be a good option.

Other MR sequences including in- and opposed-phase spoiled gradient-recalled echo T1-weighted imaging and contrast-enhanced fat-suppressed three-dimensional spoiled gradient-recalled echo imaging were performed.

### 2.3. Reference Standard

Standard of reference for FLL detection was established by the consensus reading of the two observers (Ye Tan and Jie Zhu, with 15 and 10 years of experience in abdominal imaging, resp.), using all available MR sequences including precontrast T1-weighted sequence, in- and opposed-phase gradient-recalled-echo T1-weighted sequence, and dynamic contrast-enhanced MR sequence. A lesion was considered as present if it could be detected on at least one sequence and also was confirmed by histopathologic analysis or follow-up MR imaging.

FLLs characterization was established optimally by histopathologic findings. For cases without available histopathologic findings, the clinical diagnosis was made by the combination of clinical history, typical MR imaging findings, and follow-up MR imaging with a minimum interval of 6 months. The clinical diagnosis of benign lesions including FNH, IPT, solitary necrotic nodule (SNN), and hepatic pseudolipoma was made by using validated criteria [24–27] and by their stable appearance at follow-up MR imaging with a minimum interval of 6 months. HCCs were diagnosed clinically from a complicated consideration of cirrhosis background, typical imaging findings [28, 29], the American Association for the Study of Liver Disease (AASLD) criteria for HCC [30], elevated tumor markers (e.g., *α*-fetoprotein), and progressively enlargement in follow-up. Metastases were diagnosed on the basis of presence of a known primary malignancy, MR imaging findings [31, 32], and follow-up imaging results showing interval progression.

### 2.4. Qualitative Evaluation

All MR images were independently interpreted by two observers (Yue Guo and Chen Zhang, with 8 and 7 years of experience in abdominal imaging, resp.) who were blinded to clinical history and imaging reports. DWI600, DWI100, and T2WI were randomly analyzed in three sessions separated by at least 3 weeks to minimize a recall bias. All the cases in each session were interpreted in a random manner. For each patient, the number, size, location (with Couinaud segments delineated), and image number of FLLs were recorded. A maximum of 5 largest lesions were recorded per patient, if multiple FLLs were present. Evaluation was done at GE ADW 4.4 workstation. Each sequence for lesion conspicuity was subjectively rated by using a four-point scale, as follows: score 1, definitely not present; score 2, probably not present; score 3, probably present; score 4, definitely present. Positive detection was calculated based on lesions assigned more than or equal to score 3.

### 2.5. Statistical Analysis

The diagnostic accuracy of DWI600, DWI100, and T2WI for solid FLLs detection was evaluated by comparing the detection rate between each two MR sequences. A statistical analysis was done by using a binary logistic regression model in which the detection accuracy was included as a dependent variable. Subgroup analyses based on the type (benign and malignant), size (≤10 mm, >10 mm), and location (right lobe and left lobe) were also performed. The cut-point of 10 mm selected was based on the average diameter of intrahepatic vessels we measured.

*k* statistic was used to assess interobserver agreement for lesion detection, defined as poor (<0.2), fair (0.21–0.40), moderate (0.41–0.60), good (0.61–0.80), and excellent (0.81–1.00) agreement. All statistical analyses were performed using SPSS 17.0 software (Windows, SPSS, Chicago, IL). A *P* value of less than 0.05 was considered to be statistically significant.

## 3. Results 

### 3.1. Consensus Reading

Ninety-seven patients had a total of 137 solid FLLs (size range, 5.5−75 mm; mean, 21.9 mm) (Table 2). Sixty patients had 96 malignant solid FLLs, including 73 HCCs, 20 metastases, two cholangiocarcinomas, and one hemangioendothelioma. Histopathologic diagnosis was used for 52 HCCs, one metastatic lesion, two cholangiocarcinomas, and one hemangioendothelioma. Clinical diagnosis was used for 21 HCCs and 19 metastases. Thirty-seven patients had 41 benign solid FLLs, including 14 FNHs, 10 IPTs, nine SNNs, five hepatic pseudolipomas, one angiomyolipoma, one hepatic adenoma, and one ectopic adrenal adenoma. Among those benign solid FLLs, one angiomyolipoma, two FNHs, one adenoma, one ectopic adrenal adenoma, two IPTs, and one hepatic pseudolipoma were diagnosed pathologically. The diagnosis of remaining benign solid FLLs was made clinically according to the standard of reference.

### 3.2. Qualitative Evaluation

**Figure 1:**
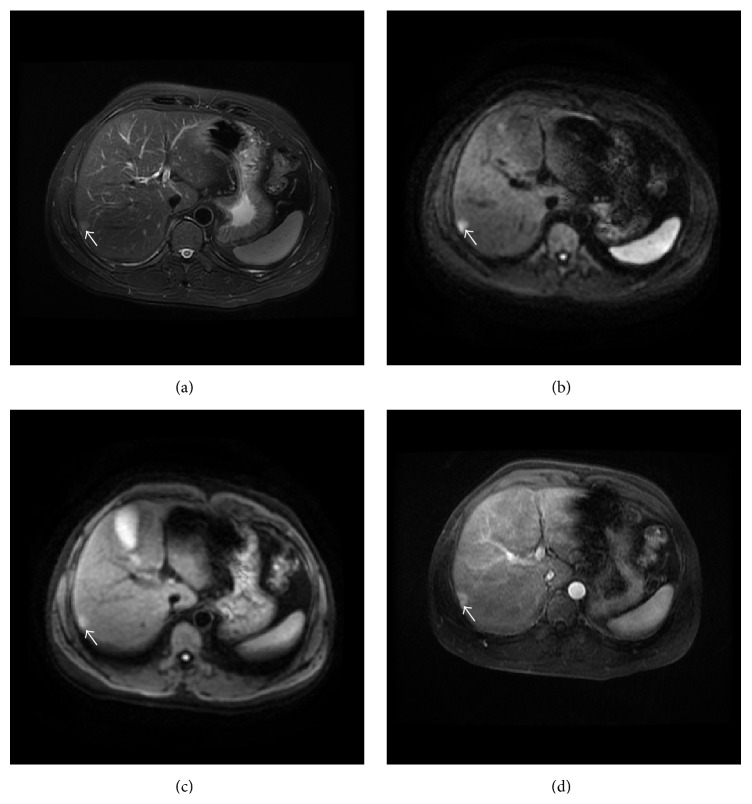
Transverse images in 49-year-old man with HCC. (a) Fat-suppressed T2-weighted fast spin-echo image shows that lesion is slightly hyperintense to the liver. (b) DW image with higher *b*-value shows that lesion is strongly hyperintense to the liver. (c) DW image with low *b*-value shows that lesion is slightly hyperintense to the liver with ill-defined margin. (d) Contrast-enhanced T1-weighted image in arterial phase demonstrates strong enhancement of lesion.

**Figure 2:**
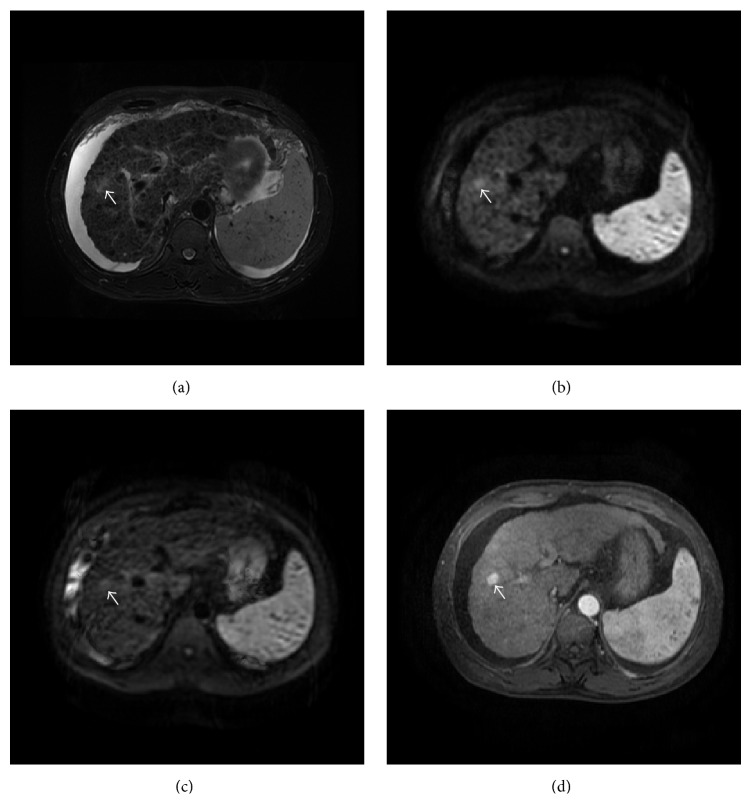
Transverse images in 62-year-old man with HCC. (a) Fat-suppressed T2-weighted fast spin-echo image shows that lesion is difficult to be detected. (b) DW image with higher *b*-value shows that lesion is strongly hyperintense to the liver. (c) DW image with low *b*-value shows that lesion is obscure. (d) Contrast-enhanced T1-weighted image in arterial phase demonstrates strong enhancement of lesion.

**Figure 3:**
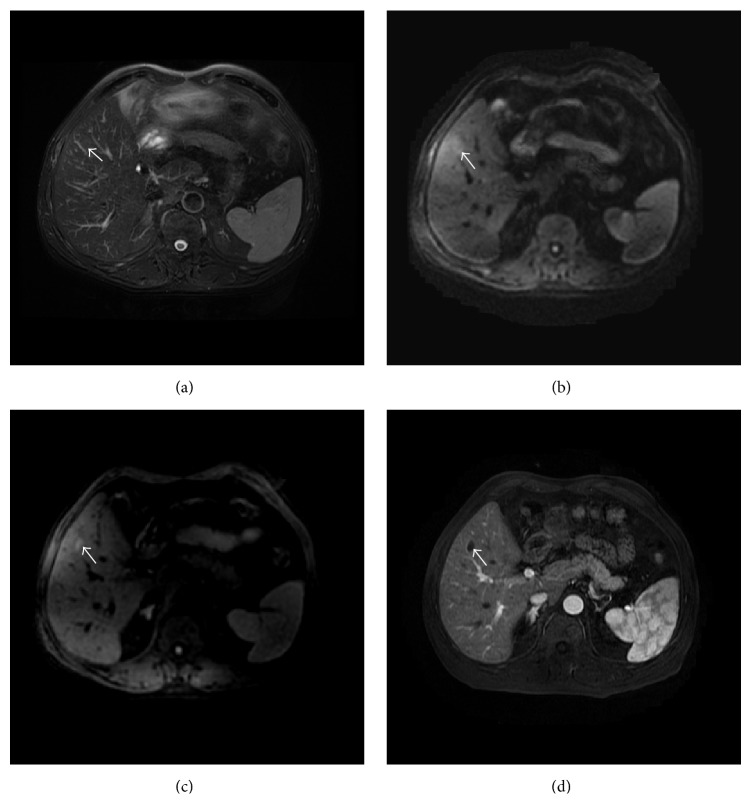
Transverse images in 56-year-old man with solitary necrotic nodule. (a) Fat-suppressed T2-weighted fast spin-echo image shows that lesion is isointense to the liver. (b) DW image with higher *b*-value shows that lesion is difficult to be identified. (c) DW image with low *b*-value shows lesion of hyperintensity could easily be detected. (d) Contrast-enhanced T1-weighted image in arterial phase demonstrates nonenhancement of lesion.

The detection rates using different MR sequences in all, benign, and malignant solid FLLs were shown in Table 3. The overall detection rate in all solid FLLs with DWI600, DWI100, and T2WI were 71.1%, 67.9%, and 62.4%, respectively. There were no significant differences between each two MR sequences. For malignant solid FLLs, DWI600 allowed identification of more FLLs (84.4%) than DWI100 (versus 72.4%, *P* < 0.05) and T2WI (versus 70.8%, *P* < 0.05) (Figures 1 and 2). The subgroup analysis in benign solid FLLs showed that the detection rate of DWI100 (57.3%) was higher than DWI600 (40.2%) and T2WI (42.7%) without significant difference (Figure 3). Meanwhile, DWI600 and T2WI were more accurate in the detection of malignant solid FLLs than of benign lesions (84.4% versus 40.2%, *P* < 0.001; 70.8% versus 42.7%, *P* < 0.05, resp.). However, no significant difference was observed for DWI100 in the detection of malignant solid FLLs in comparison of benign lesions (*P* = 0.082).

The detection rates using different MR sequences by each observer were shown in Table 4. Interobserver agreement for the FLLs detection using DWI600, DWI100, and T2WI was excellent, given the *k* values of 0.982, 0.900, and 0.861, respectively.

The subgroup analysis by lesion size showed that three MR sequences were more accurate in detection of lesions (>10 mm) than of lesions smaller than 10 mm (*P* < 0.001, for all MR sequences) (Table 5). However, there were no significant differences between each two MR sequences in detection of lesion no matter larger or smaller than 10 mm.

The subgroup analysis by lesion location showed there were no significant differences between each two MR sequences in detection of lesions in left lobe or in right lobe (Table 5). However, T2WI was significantly better in detection of FLLs in the left lobe than in the right lobe (76.8% versus 56.3%, *P* < 0.05).

### 3.3. Missed FLLs

The missed FLLs by both observers at DWI600 included 13 HCCs, eight FNHs, five pseudolipomas, six IPTs, two metastases, four SNNs, and one hepatic adenoma.

The missed FLLs by both observers at DWI100 included 20 HCCs, five FNHs, five pseudolipomas, four IPTs, four metastases, one SNN, one hepatic adenoma, and one cholangiocarcinoma.

The missed FLLs by both observers at T2WI included 22 HCCs, seven FNHs, four pseudolipomas, four IPTs, four metastases, five SNNs, and one hepatic adenoma.

## 4. Discussion

Our study showed that there were no significant differences among DWI600 (71.1%), DWI100 (67.9%), and T2WI (62.4%) in detection of solid FLLs overall. However, DWI600 (84.4%) was significantly better than DWI100 (versus 72.4%, *P* < 0.05) and T2WI (versus 70.8%, *P* < 0.05) in detection of malignant solid FLLs. It is well known that DWI with low *b*-value of approximately 100–150 sec/mm^2^ is hypothesized to attenuate signal from microcirculatory perfusion, while DWI with higher *b*-value (*b* > 500 s/mm^2^) is thought to reveal restriction of water molecular diffusion in lesions [11–13]. With the highly cellular tissue, the tortuosity of the extracellular space, and the high density of cell membranes, malignant FLLs can be more easily detected on DWI600 than on DWI100. In addition, T2WI was poorly reliable in detection of malignant solid FLLs when it showed iso- or slightly hypersignal intensity to liver parenchyma. Several studies [18, 33] have shown the limitation of T2WI in the detection of HCC in cirrhosis, mostly related to HCC signal intensity on T2WI images with appearing iso- or hypointense in 42.1%–53% of HCC. Results of our studies were in line with the findings of previous reports with lower rate of 32.2% (23.5/73, average between two observers) HCCs defined as undetectable (score < 3) according to the subjective rate system on T2WI, in comparison with 17.8% (13/73, average between two observers) HCCs undetectable on DWI600.

In addition, our study showed that DWI600 (40.2%) and T2WI (42.7%) were inferior in detecting benign solid FLLs in comparison with DWI100 (57.3%), although the differences did not reach significance. In our study, four FNHs, three SNNs, and two IPTs by observer 1 and three FNHs, three SNNs, and one IPT by observer 2 were visible on DWI100 but not on DWI600. The difference between DWI100 and DWI600 in detection of benign lesions is possibly attributed to three factors. First, over half of benign solid FLLs in our study did not show diffusion restriction. Second, although some benign solid lesions showed high signal intensity on DWI600 due to restricted water molecular diffusion, the lesion-to-liver contrast of those lesions on DWI600 was not as high as malignant lesions such as HCC [34]. Third, black blood effect, better imaging quality, and better contrast-to-noise ratio with DWI100 result in the better lesion conspicuity [12, 13]. Unfortunately, not enough benign solid FLLs as much as malignant lesions were included in our study; the potential benefit of DWI100 in detection of benign solid FLLs needs more studies to be investigated.

Of interest, our study found DWI600 had the higher detection rate of FLLs on left lobe than on right lobe. Several studies [21, 35] reported the poor visibility of the left lobe on DWI due to cardiac-motion induced signal loss; DWI was thought to be less sensitive in the left lobe. However, one must note that lesions with definite diffusion restriction can also be detected on DWI600 in left lobe. Meanwhile, results from our subgroup analysis on lesion size suggested that the detection rate was significantly influenced by the lesion size. The larger the lesion was, the easier it could be detected on all three MR sequences.

Our results were in disagreement with previous studies [14, 17] that DWI with a low *b*-value can significantly improve detection rate of both malignant and benign FLLs, comparing with T2-weighted imaging. For example, our study found that the detection rate of T2WI (70.8%) in malignant solid FLLs was as much as of DWI100 (72.4%). There is no doubt that the suppression of intrahepatic vessels with DWI100 considerably improves the detectability of perivascular lesions. However, T2WI is still helpful in detecting perivascular lesions when the size of lesion is much larger than adjacent vessel or when the lesion-to-vessel contrast is obvious. In addition, one potential explanation is that lacking the susceptibility artifact and image distortion, T2WI is helpful in detection of FLLs in the periphery of the liver and in the subphrenic hepatic areas.

There are several limitations of the study. First, the low and higher *b*-value DW images were acquired using different techniques for respiratory motion suppression and different numbers of signal averages. The results of this study may be confounded by the differences in acquisition between breath-hold and respiratory-triggered study and differences in the number of signal averages. The reason is that the MR scanners used in our study could not obtain two *b*-values DWI in one acquisition. The breath-hold acquisition mode for the DWI100 was selected to achieve a short acquisition time and few motion artifacts. In addition, the superiority of respiratory-triggered DW imaging over breath-hold DW imaging for lesion detection has been suggested by previous report [14]. Second, not all FLLs were confirmed pathologically. However, clinical diagnosis can be firmly established based on careful consensus reading by experienced abdominal radiologists and follow-up data. Third, we did not make analysis of apparent diffusion coefficient (ADC) maps, because analysis of ADC values was not within the scope of this work. Fourth, we did not investigate contrast-enhanced MR sequence in our study. Contrast-enhanced MR sequence is better than unenhanced MR sequences in the detection of FLLs [36]. Nevertheless, DWI and T2WI play an irreplaceable role in the detection of FLLs in patients having contrast agent allergy or renal dysfunction. Fifth, the number of benign FLLs and small FLLs (<10 mm) was relatively small. However, we believe our results are valid because we included a consecutive series of patients during a relatively long period and covered topic of common disease.

In conclusion, DWI600 was equivalent to DWI100 and T2WI for the detection of solid FLLs in all lesions but was significantly more accurate in detection of malignant solid FLLs and lesions larger than 10 mm. The results of our study show the superiority of DWI600 in the detection of malignant solid FLLs, but also the disadvantage of DWI600 for the depiction of benign solid FLLs such as FNHs and IPTs and tiny lesions. Both low and higher *b*-value diffusion-weighted imaging should be recommended as supplementary MR sequences in clinical practice.

## Figures and Tables

**Table 1:** Parameters of three evaluation MR sequences.

Parameters	DWI600	DWI100	T2WI
Acquisition mode	Respiratory-triggered	Breath-hold	Respiratory-triggered
Repetition time (TR)	2-3 respiratory cycles	2400 ms	2-3 respiratory cycles
Echo time (TE)	Minimum	Minimum	80 ± 10 ms
*b*-value (s/mm^2^)	0 and 600	0 and 100	/
Section thickness (mm)	6	6	6
Intersection gap (mm)	1.5	1.5	1.5
Field of view (mm)	320–380	320–380	320–380
Rectangle FOV	90%–100%	90%–100%	75%
Matrix	128 × 128	128 × 128	224 × 320
Number of signal averages	4	2	2
Parallel acceleration factor	2	2	/
Echo train length	64	64	<20
Acquisition time	2-3 min	19 s	3–5 min

DWI600: diffusion-weighted imaging with *b*-value of 0, 600 sec/mm^2^; DWI100: diffusion-weighted imaging with *b*-value of 0, 100 sec/mm^2^; T2WI: T2-weighted imaging.

**Table 2:** Clinical information of the 97 patients and characteristics of 137 solid FLLs.

Age range (mean age)	25–77 years (52.2 years)
Sex (M/F)	65/32
Background liver	30 chronic hepatitis 25 liver cirrhosis 20 steatosis 22 normal liver
Diagnosis of the lesions (*n* = 137)	
Benign (*n* = 41)	14 FNHs 10 IPTs 9 SNNs 5 hepatic pseudolipomas 1 angiomyolipoma 1 hepatic adenoma 1 ectopic adrenal adenoma
Malignant (*n* = 96)	73 HCCs 20 metastases 2 cholangiocarcinomas 1 hemangioendothelioma
Location of the lesions	41 left lobes and 96 right lobes
Primary site of malignancy Patients (*n* = 5)	3 rectal-colons 1 breast 1 pancreas

FNH: focal nodular hyperplasia; IPT: inflammatory pseudotumor; SNN: solitary necrotic nodule; HCC: hepatocellular carcinoma.

**Table 3:** Detection rate of solid FLLs with each sequence in all, benign, and malignant lesions.

Sequence		All lesions (*n* = 137)	Benign lesions (*n* = 41)	Malignant lesions (*n* = 96)
DWI600		71.1 (97.5/137)	40.2 (16.5/41)	84.4 (81/96)
DWI100		67.9 (93/137)	57.3 (23.5/41)	72.4 (69.5/96)
T2WI		62.4 (85.5/137)	42.7 (17.5/41)	70.8 (68/96)
*P* value	DWI100 versus DWI600	0.573	0.127	0.04
	DWI100 versus T2WI	0.333	0.190	0.838
	DWI600 versus T2WI	0.125	0.825	0.024

Data are averaged for two independent observers. Unless otherwise indicated, numbers are percentages, with raw data in parentheses. DWI600: diffusion-weighted imaging with *b*-value of 0, 600 sec/mm^2^; DWI100: diffusion-weighted imaging with *b*-value of 0, 100 sec/mm^2^; T2WI: T2-weighted imaging.

**Table 4:** Detection rate of solid FLLs with each sequence by the two readers in all, benign, and malignant lesions.

	Sequence	All lesions (*n* = 137)	Benign lesions (*n* = 41)	Malignant lesions (*n* = 96)
Observer 1	DWI600	71.5 (98/137)	41.4 (17/41)	84.4 (81/96)
	DWI100	69.3 (95/137)	60.9 (25/41)	72.9 (70/96)
	T2WI	59.1 (81/137)	36.6 (15/41)	68.8 (66/96)

Observer 2	DWI600	70.8 (97/137)	39.0 (16/41)	84.4 (81/96)
	DWI100	66.4 (91/137)	53.7 (22/41)	71.9 (69/96)
	T2WI	65.7 (90/137)	48.8 (20/41)	72.9 (70/96)

Unless otherwise indicated, numbers are percentages, with raw data in parentheses. DWI600: diffusion-weighted imaging with *b*-value of 0, 600 sec/mm^2^; DWI100: diffusion-weighted imaging with *b*-value of 0, 100 sec/mm^2^; T2WI: T2-weighted imaging.

**Table 5:** Detection rate of solid FLLs with each sequence stratified by location and size.

Sequence		Lesion location		Lesion size	
		Left lobe (*n* = 41)	Right lobe (*n* = 96)	≤10 mm	>10 mm
DWI600		79.3 (32.5/41)	67.7 (65/96)	31.2 (7.5/24)	79.6 (90/113)
DWI100		70.7 (29/41)	66.7 (64/96)	25 (6/24)	77.0 (87/113)
T2WI		76.8 (31.5/41)	56.3 (54/96)	16.7 (4/24)	72.1 (81.5/113)
*P* value	DWI100 versus DWI600	0.411	0.878	0.588	0.628
	DWI100 versus T2WI	0.573	0.138	0.477	0.382
	DWI600 versus T2WI	0.794	0.102	0.212	0.175

Data are averaged for two independent observers. Unless otherwise indicated, numbers are percentages, with raw data in parentheses. DWI600: diffusion-weighted imaging with *b*-value of 0, 600 sec/mm^2^; DWI100: diffusion-weighted imaging with *b*-value of 0, 100 sec/mm^2^; T2WI: T2-weighted imaging.
